# Prenatal Dexamethasone and Postnatal High-Fat Diet Decrease Interferon Gamma Production through an Age-Dependent Histone Modification in Male Sprague-Dawley Rats

**DOI:** 10.3390/ijms17101610

**Published:** 2016-09-22

**Authors:** Hong-Ren Yu, You-Lin Tain, Jiunn-Ming Sheen, Mao-Meng Tiao, Chih-Cheng Chen, Ho-Chang Kuo, Pi-Lien Hung, Kai-Sheng Hsieh, Li-Tung Huang

**Affiliations:** Department of Pediatrics, Chang Gung Memorial Hospital-Kaohsiung Medical Center, Graduate Institute of Clinical Medical Science, Chang Gung University College of Medicine, Kaohsiung 833, Taiwan; yuu2004taiwan@yahoo.com.tw (H.-R.Y.); tainyl@hotmail.com (Y.-L.T.); ray.sheen@gmail.com (J.-M.S.); pc006581@yahoo.com.tw (M.-M.T.); charllysc@yahoo.com.tw (C.-C.C.); erickuo48@yahoo.com.tw (H.-C.K.); flora1402@cgmh.org.tw (P.-L.H.); kshsieh@adm.cgmh.org.tw (K.-S.H.)

**Keywords:** prenatal glucocorticoid, high-fat diet, IFN-γ, histone modification, age-dependent

## Abstract

Overexposure to prenatal glucocorticoid (GC) disturbs hypothalamic-pituitary-adrenocortical axis-associated neuroendocrine metabolism and susceptibility to metabolic syndrome. A high-fat (HF) diet is a major environmental factor that can cause metabolic syndrome. We aimed to investigate whether prenatal GC plus a postnatal HF diet could alter immune programming in rat offspring. Pregnant Sprague-Dawley rats were given intraperitoneal injections of dexamethasone or saline at 14–21 days of gestation. Male offspring were then divided into four groups: vehicle, prenatal dexamethasone exposure, postnatal HF diet (VHF), and prenatal dexamethasone exposure plus a postnatal HF diet (DHF). The rats were sacrificed and adaptive immune function was evaluated. Compared to the vehicle, the DHF group had lower interferon gamma (IFN-γ) production by splenocytes at postnatal day 120. Decreases in H3K9 acetylation and H3K36me3 levels at the IFN-γ promoter correlated with decreased IFN-γ production. The impaired IFN-γ production and aberrant site-specific histone modification at the IFN-γ promoter by prenatal dexamethasone treatment plus a postnatal HF diet resulted in resilience at postnatal day 180. Prenatal dexamethasone and a postnatal HF diet decreased IFN-γ production through a site-specific and an age-dependent histone modification. These findings suggest a mechanism by which prenatal exposure to GC and a postnatal environment exert effects on fetal immunity programming.

## 1. Background

Prenatal glucocorticoid (GC) exposure has been shown to effectively stimulate the development of the fetal lung and decrease mortality and morbidity after birth [1]. However, overexposure to GC has been observed in prenatal stress, which can change the homeostasis of lipid and glucose metabolism in later life [2,3]. In humans, the modulating effect of prenatal GC on the developing immune system has been evaluated in several small series. In these limited studies on neonates to infants, prenatal exposure to GC seemed to have only a limited effect on the developing immune system [4,5,6]. However, prolonged adverse effects on the immune system after prenatal GC treatment have been observed. We previously found that prenatal GC treatment reduced the capacity of adolescent rat splenocytes to produce tumor necrosis factor alpha (TNF-α) rather than interferon gamma (IFN-γ), and that prenatal GC treatment seemed to have a more profound effect on the innate immune system than the adaptive immune system [7]. However, prolonged adverse effects on the immune system after prenatal GC treatment have not been observed.

The adaptive immune system is very important for host defense against pathogens. The adaptive immune system is superior to the innate immune system for its specificity, memory, and flexibility to respond to a diversity of microorganisms. Helper T-cell (Th) immunity, central to the adaptive immune, can be characterized as Th1, Th2, and Th3 cells by their cytokine profile [8,9]. Th1 cells produce interleukin (IL)-2 and IFN-γ. Th2 cells produce IL-4, IL-5, and IL-13 [10]. Th3 cells produce IL-10 and TGF-β. Th1 is responsible for defense against intracellular microorganisms. Th2 promotes allergic reaction and is also activated in response to parasite infections [10]. The function of Th1 and Th2 cells is regulated by regulatory T (Th3; Treg) cells. Treg cells are important in the maintenance of peripheral tolerance [8,9]. We previously found that prenatal GC treatment reduced the capacity of adolescent rat splenocytes to produce TNF-α rather than IFN-γ, and that prenatal GC treatment seemed to have a more profound effect on the innate immune system than the adaptive immune system [7].

Obesity has become a major problem in many developed countries and has been linked with many health problems. Clinical and experimental data suggest that changes in immune function as a result of obesity may lead to susceptibility to infection [11,12]. Epidemiological studies have also reported a higher prevalence of atopic diseases and asthma in obese subjects than in those with a normal body weight [13,14]. Studies on cytokine production in obese patients have reported conflicting results. Increased IFN-γ and IL-4 production has been observed in obese mice splenocytes upon stimulation with mitogen, whereas decreased IFN-γ production has been observed in human mononuclear cells stimulated with phorbol myristate acetate (PMA) [12,15]. Offspring of rats from stressed dams were more susceptible to high fat (HF) diet induced-obesity, and this susceptibility was thought to be due to excessive exposure of the developing fetus to maternal GC, although disagreement exists [16]. Although prenatal GC exposure affects the innate immune system [7], whether prenatal GC exposure plus a postnatal HF diet can disturb adaptive immune function is unknown. The aim of this study was therefore to investigate whether prenatal GC plus a postnatal HF diet can induce adaptive immune programming in the offspring of rats.

In the present study, we established a prenatal rat GC stress model as in our previous study [7]. A post-weaning HF diet was given to induce the production of metabolic syndrome and immune programming in the offspring. The results showed that prenatal exposure to GC plus a HF diet induced age-dependent histone modification, leading to impaired IFN-γ production. This model clearly showed aggravation of adaptive immune function caused by prenatal GC exposure plus a postnatal HF diet. This present study may help to clarify the mechanism by which prenatal exposure and the postnatal environment exert effects on fetal immunity programming.

## 2. Results

### 2.1. Postnatal High-Fat (HF) Diet Increased Body Weight (BW) but Decreased the Spleen:BW Ratio

As expected, a postnatal HF diet resulted in a heavier BW than exposure to the vehicle and dexamethasone (Table 1). As previously reported, the DEX group had a higher spleen weight-to-BW ratio than the vehicle group at D120. In this study, we further found that postnatal HF exposure resulted in a lower spleen weight-to-BW ratio than in the vehicle group. The DEX group had a higher thymus weight-to-BW ratio than the vehicle group at D120. The VHF and DHF groups also showed a higher thymus weight-to-BW ratio than the VEH group at D120, although this did not reach statistical significance. At D180, although the VHF and DHF groups showed a heavier thymus weight and spleen weight, respectively, than the VEH group, this did not reach statistical significance after being corrected with BW. In contrast to postnatal HF exposure, the DEX group showed a lower thymus weight-to-BW ratio than the vehicle group at D180. These results suggested, relative to postnatal HF exposure, that prenatal GC exposure has the greater impact on thymus development. These results also implied the immunological programming impacts of prenatal GC and postnatal HF exposures are in an age-dependent manner.

### 2.2. Both Prenatal Dexamethasone Treatment and Postnatal HF Diet Changed the Leukocyte Subsets of the Rats at the Adolescent Stage

There were no significant differences between the VEH, DEX, VHF, and DHF groups in terms of total leukocyte count, red blood cell count, and platelet count at D120. In the leukocyte subsets, the DEX, VHF, and DHF groups all had higher neutrophil-to-lymphocyte ratios than the VEH group at D120. This showed that both prenatal dexamethasone treatment and a postnatal HF diet resulted in immunotoxicity in the developing rats (Table 2). Flow cytometry with the indicated antibodies (CD3, CD4, CD8a, CD4/8a ratio, and CD45RA) was used to analyze the lymphocyte sub-populations (Figure S1). The VHF group had higher levels of CD4 and CD8a double positive cells and higher levels of CD4 and CD8a double negative cells than the VEH group. The DHF group only had higher levels of CD4 and CD8a double negative cells than the VEH group at D120 (Table 2). At D180, the total blood leukocyte count of the DHF group was lower than the VEH group. However, the altered neutrophil/lymphocyte ratio, CD4CD8a DP, or DN cells in DEX, VHF, and DHF seen in D120 were all recovered at D80.

### 2.3. Prenatal Dexamethasone Treatment Plus a Postnatal HF Diet Changed Rat Spleen Innate Cytokine Production at D120

**Figure 1:**
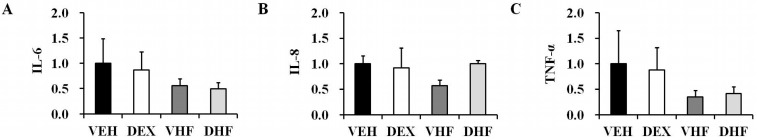
Quantitative reverse transcription-polymerase chain reaction (RT-PCR) analysis of innate cytokine mRNA expressions in the rat spleens at the adolescent stage. Representative RT-PCR analysis of (**A**) IL-6; (**B**) IL-8; and (**C**) TNF-α mRNA levels of the spleens of the four groups at post-natal day 120 (D120) (PPIB as internal control gene) (*n =* 5 or 6 for each group). Abbreviations: VEH; vehicle: DEX; prenatal dexamethasone exposure: VHF; postnatal high-fat diet exposure: DHF; prenatal dexamethasone plus postnatal high-fat diet exposure.

**Figure 2:**
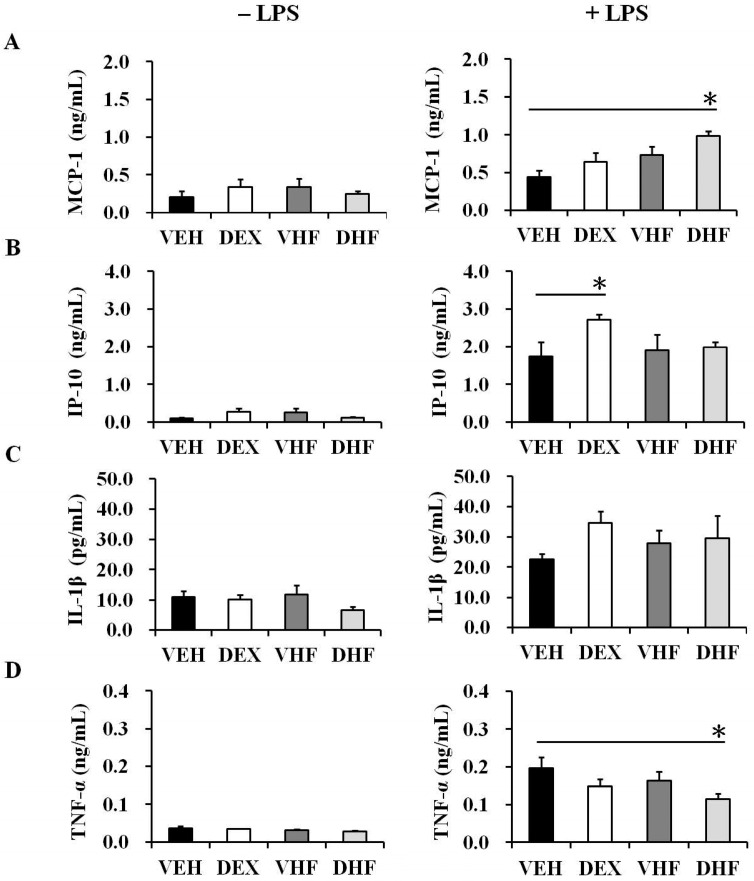
Effects of prenatal dexamethasone/postnatal high-fat diet treatment on pro-inflammatory mediator production in rat splenocytes. Isolated splenocytes from the VEH, DEX, VHF, and DHF groups at D120 were suspended at 2 × 10^6^/mL in 24-well plates then treated with 100 ng/mL of LPS. The culture supernatants were collected at 24 h and then levels of (**A**) MCP-1; (**B**) IP-10; (**C**) IL-1β; and (**D**) TNF-α were detected using a Multiplex Assay system or ELISA, respectively (*n =* 5 or 6 for each group). * *p* < 0.05.

To investigate innate immunity-related cytokines, the transcript expression levels of IL-6, IL-8, and TNF-α in the rat spleens were assessed (Figure 1). In order to find a reliable reference gene for transcript analysis, we tried to investigate the mRNA expressions of PPIB, beta-actin, and GAPDH. We found that PPIB showed less variation than beta-actin and GAPDH in rat spleen tissue (Figure S2). PPIB as a reliable reference gene in rat spleen mRNA analysis was compatible with another previous report [17]. Thereafter, we used PPIB as a reference gene in subsequent studies. A postnatal HF diet (VHF group) and prenatal exposure to dexamethasone plus a postnatal HF diet (DHF group) resulted in a trend of decreased IL-6 and TNF-α mRNA expressions in the rat spleens, although this did not achieve statistical significance. In another experiment, splenocytes were treated with/without LPS (100 ng/mL) for one day, and the supernatants were collected for IP-10, MCP-1, IL-1β, and TNF-α measurement. There were only a few innate cytokines produced by cultured splenocytes without LPS stimulation (Figure 2). The DEX group showed higher IP-10 production than the VEH group (Figure 2B). The DHF group showed higher MCP-1 but lower TNF-α production than the VEH group (Figure 2B,D).

### 2.4. Postnatal HF Diet Decreased the Proliferation of Rat Splenocytes at the Adolescent Stage

**Figure 3:**
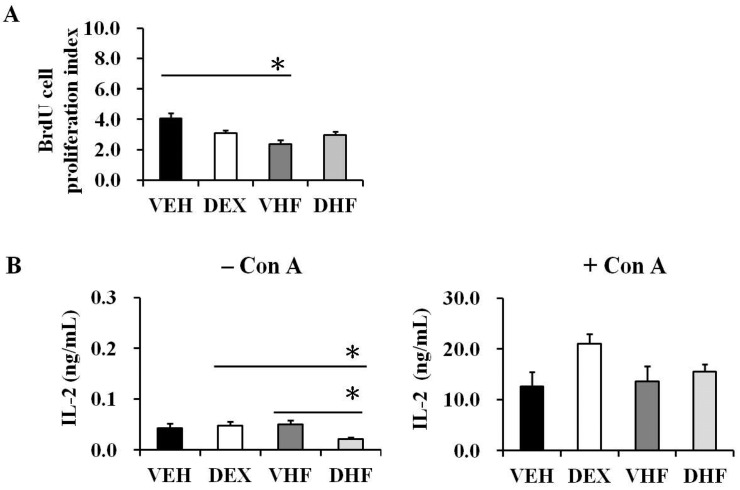
Effects of prenatal dexamethasone/postnatal high-fat diet treatment on rat splenocyte proliferation and IL-2 productions at D120. (**A**) Isolated splenocytes were treated with ConA, and splenocyte proliferation was assayed using BrdU assays as described in the Materials and Methods section. The proliferation index was presented as the ratio of optical density of ConA stimulation/optical density of phosphate buffered saline stimulation. The data shown are mean ± SE from five or six replicate experiments (* *p* < 0.05 compared with the VEH group); (**B**) For IL-2 production, splenocytes were suspended at 2 × 10^6^/mL in 24-well plates and then treated with 5 μg/mL of ConA. The culture supernatants were collected at 72 h and then IL-2 was detected using a Multiplex Assay system (Millipore).

Upon ConA stimulation, the VHF group showed a decrease in the proliferation of splenocytes at D120 (Figure 3A). This decrease seemed to be IL-2 independent, because there was no statistically significant difference in IL-2 production between the VHF and other groups with ConA stimulation (Figure 3B). The splenocytes produced only a limited amount of IL-2 without mitogen stimulation.

### 2.5. Prenatal Dexamethasone Exposure Plus a Postnatal HF Diet Suppressed Rat Splenocyte IFN-γ Production at D120

**Figure 4:**
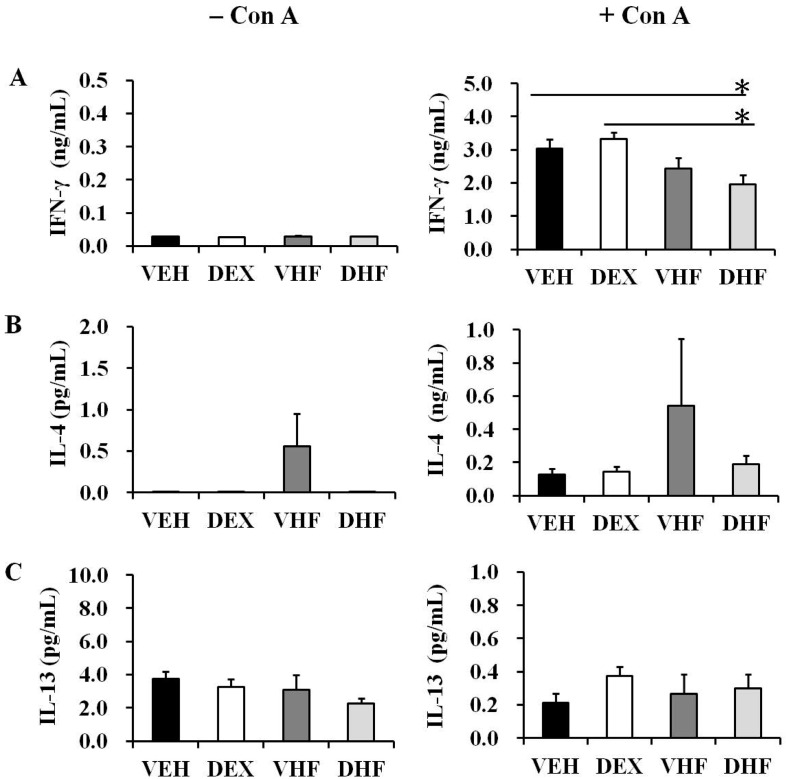
Effects of prenatal dexamethasone/postnatal high-fat diet treatment on Th1/Th2 cytokine production in rat splenocytes at D120. Isolated splenocytes from the VEH, DEX, VHF, and DHF groups at D120 were suspended at 2 × 10^6^/mL in 24-well plates and then treated with 5 μg/mL of ConA. The culture supernatants were collected at 72 h and then levels of (**A**) IFN-γ; (**B**) IL-4; and (**C**) IL-13 were detected (*n =* 5 or 6 for each group). * *p* < 0.05.

**Figure 5:**
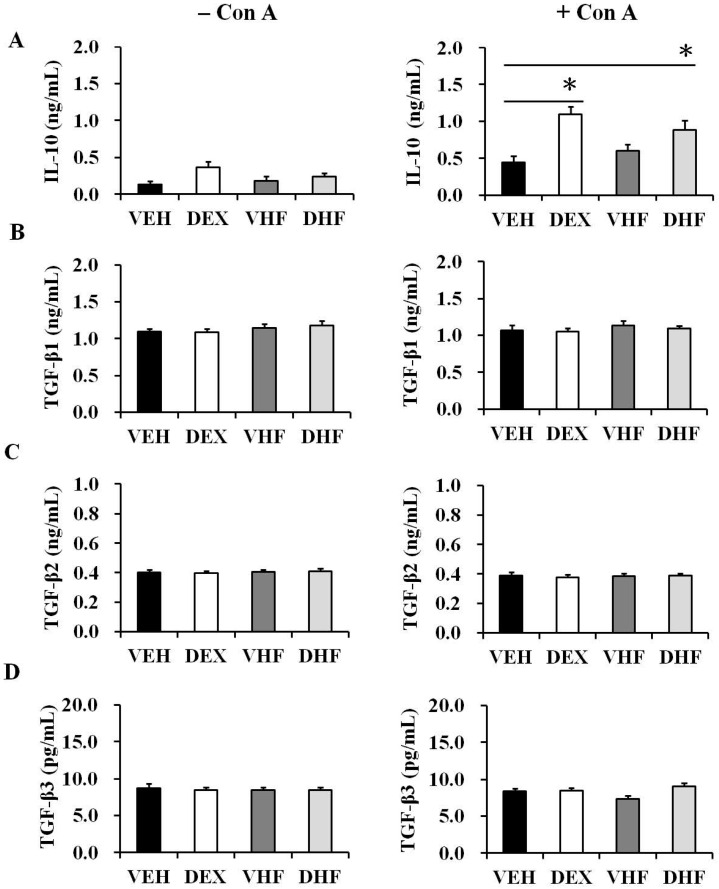
Effects of prenatal dexamethasone/postnatal high-fat diet treatment on Th3 cytokine production in rat splenocytes at D120. Isolated splenocytes from the VEH, DEX, VHF and DHF groups at postnatal day 120 were treated with 5 μg/mL of ConA. The culture supernatants were collected at 72 h and then levels of (**A**) IL-10; (**B**) TGF-β1; (**C**) TGF-β2; and (**D**) TGF-β3 were detected (*n =* 5 or 6 for each group). * *p* < 0.05.

The effect of prenatal dexamethasone exposure plus postnatal HF diet on adaptive immunity-related cytokines was subsequently investigated. Splenocytes underwent ConA stimulation for three days, and supernatants were collected to measure cytokine concentrations. Only prenatal dexamethasone or only postnatal high fat exposure did not affect IFN-γ production at D120 (Figure 4A). In contrast, the DHF group had lower IFN-γ production at D120 than the VEH and DEX groups. We also evaluated the influence of prenatal dexamethasone exposure and/or postnatal HF diet on the production of Th2-related cytokines (IL-4, IL-5, and IL-13). IL-5 production was not analyzed, as it was undetectable in several rats. Two rats in the VHF group had high IL-4 production; however, there was no statistical significance compared with the other groups (Figure 4B). Prenatal dexamethasone exposure and/or prenatal HF diet did not influence IL-13 production (Figure 4C). The DEX and DHF groups had higher IL-10 production than did the VEH and VHF groups at D120 (Figure 5A), suggesting that the increase in IL-10 production resulted from exposure to prenatal dexamethasone. Among the TGF-β cytokines, TGF-β1 had the highest level, followed by TGF-β2. TGF-β3 production by the rat splenocytes was much lower than TGF-β1 or TGF-β2. Prenatal dexamethasone exposure and/or prenatal HF diet did not affect TGF-β1, TGF-β2, or TGF-β3 production at D120 (Figure 5).

**Figure 6:**
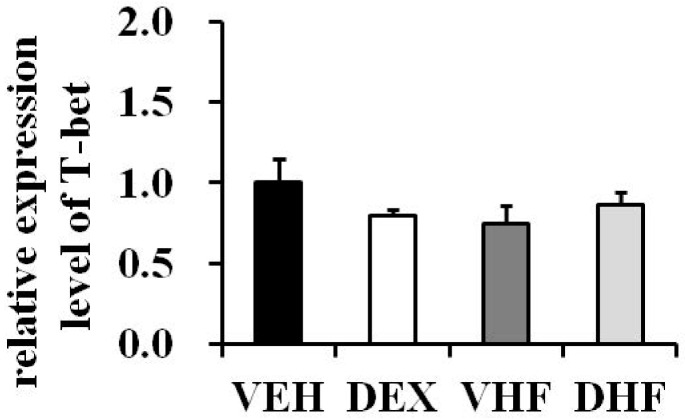
Quantitative reverse transcription-polymerase chain reaction (RT-PCR) analysis of Th1-related transcription factor T-bet mRNA expression in rat spleens at D120. T-bet mRNA levels in rat spleen tissues are relative to the VEH group at post-natal day 120 (PPIB as internal control gene) (*n =* 5 or 6 for each group).

The next experiment was conducted to see whether the IFN-γ production decrease in the DHF group is determined by the Th1-related transcription factor, T-bet. As shown in Figure 6, there were no differences in T-bet mRNA expressions among the four groups.

### 2.6. Prenatal Dexamethasone Exposure Plus Postnatal HF Diet-Induced IFN-γ Decrease at D120 Was Associated with Histone H3 Modification

**Figure 7:**
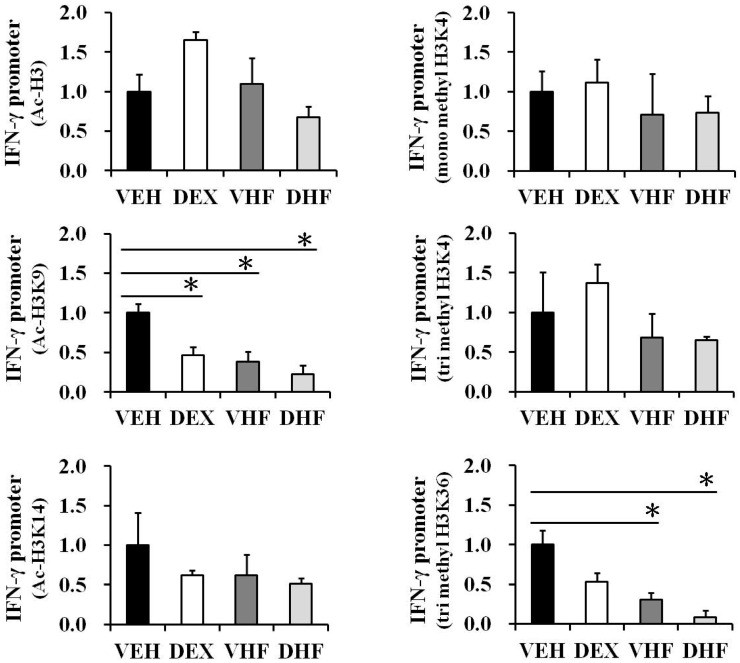
Histone H3 lysine acetylation and methylation levels at the IFN-γ promoter of the spleens with/without the indicated treatment at D120. Bar graphs showing the total H3 lysine, H3K9 and H3K14 acetylation, H3K4me1, H3K4me3, and H3K36me3 levels of the indicated treatment group relative to the vehicle group at the IFN-γ promoter. ChIP assays were performed as described in the Materials and Methods section. The results are expressed as fold difference over the vehicle (mean ± SEM; * *p* < 0.05; *n* = 3 to 5 for each group).

We previously showed that prenatal dexamethasone treatment changed TNF-α production by rat splenocytes partly though histone modification [7]. In the current study, we investigated the effect of prenatal dexamethasone exposure and/or postnatal HF diet on IFN-γ promoter histone modification. ChIP assays were performed on the spleen nuclear extracts of four-month-old rats using antibodies directed against acetyl-histone H3 (total Lys sites), acetyl-histone H3K9 (Lys 9), and acetyl-histone H3K14 (Lys 14). Quantitative real-time PCR analysis was used to determine the percentage of IFN-γ promoter input DNA that was bound to these proteins. We found that the IFN-γ promoter (within 400 bp upstream of the exon) was responsible for a significant decrease in acetylation in histone H3K9 after prenatal dexamethasone exposure plus postnatal HF diet (Figure 7). The enrichment of H3K9 acetylation on the IFN-γ promoter of the DHF group was 0.33 ± 0.12-fold relative to the VEH group. Furthermore, the DHF group had the lowest H3K9 acetylation modified level on the IFN-γ promoter among the four groups, although there was no significant difference compared with the VHF group.

We then investigated whether promoter histone H3 lysine methylation played a role in the prenatal DEX-related transcriptional decrease in IFN-γ in rat spleens. ChIP assays were performed on the nuclear extracts using antibodies directed against H3K4me1, H3K4me3, and H3K36me3. We found that postnatal HF diet and prenatal dexamethasone exposure plus postnatal HF diet also resulted in a decrease of H3K36me3 enrichment on the IFN-γ promoter compared to the VEH group (Figure 7). The H3K36me3 modified levels at the IFN-γ promoters of the VHF and DHF groups were 0.33 ± 0.09- and 0.26 ± 0.10-fold relative to the VEH group, respectively. The DHF group also had the lowest H3K36me3 modified level at the IFN-γ promoter among the four groups. These decreases in H3K9 acetylation and H3K36me3 levels at the IFN-γ promoter were correlated with a decreased expression of IFN-γ (Figure 4A).

### 2.7. The Impact of Prenatal Dexamethasone Exposure plus Postnatal HF Diet on IFN-γ Production by Rat Splenocytes at D120 Was Diminished at D180

**Figure 8:**
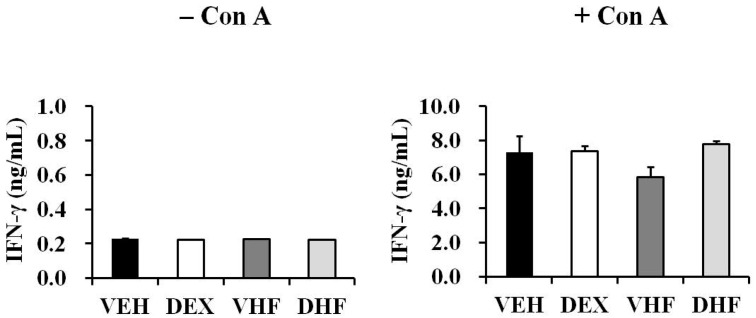
Effects of prenatal dexamethasone/postnatal high-fat diet treatment on IFN-γ production by rat splenocytes at D180. Isolated splenocytes from the VEH, DEX, VHF, and DHF groups at D180 were suspended at 2 × 10^6^/mL in 24-well plates and then treated with 5 μg/mL of ConA. The culture supernatants were collected at 72 h and then the level of IFN-γ was detected (* *p* < 0.05; *n* = 3 to 5 for each group).

In this study, the rats were sacrificed at seven days, 120 days, or 180 days after birth. These time points were considered to be equivalent to newborn, young adult, and middle-age stages in humans [18,19,20] and were chosen in order to investigate whether the suppressive effect of prenatal dexamethasone exposure plus postnatal HF diet on IFN-γ production persisted to an older age. Upon ConA stimulation, the splenocytes of the D180 rats produced more IFN-γ than the D120 rats (D180 VEH vs. D120 VEH: 7284.3 ± 949.6 vs. 3028.3 ± 270.8 ng/mL; *p =* 0.015; Figure 8). However, prenatal dexamethasone exposure plus a postnatal HF diet did not suppress IFN-γ production at D180. This suggested that the impact of prenatal dexamethasone exposure on IFN-γ production decreased with age.

### 2.8. Histone Modification of the IFN-γ Promoter Showed Age-Dependent Dynamic Changes Compatible with IFN-γ Production

**Figure 9:**
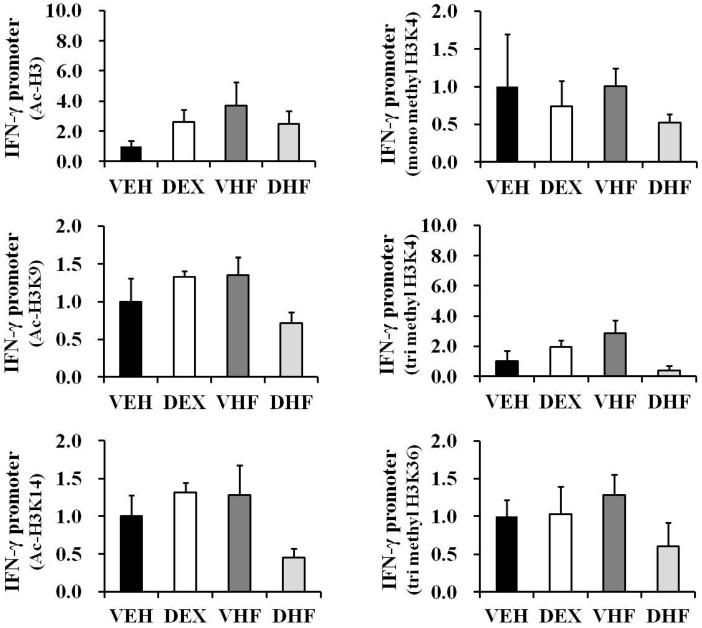
Histone H3 lysine acetylation and methylation levels at the IFN-γ promoter of spleens with/without the indicated treatment at D180. Bar graphs show the total H3 lysine, H3K9 and H3K14 acetylation, H3K4me1, H3K4me3, and H3K36me3 levels of the indicated treatment groups relative to the vehicle at the IFN-γ promoter. ChIP assays were performed as described in the Materials and Methods section. The results were expressed as fold difference over the vehicle (mean ± SEM; *n* = 3 for each group).

Since the impact of prenatal dexamethasone exposure plus postnatal HF diet on IFN-γ production by rat splenocytes was diminished at D180, ChIP assays were performed to investigate whether the histone modifications of the IFN-γ promoter changed with age. There were no significant differences in histone H3 acetylation (total H3, H3K9 and H3K14) and histone H3 lysine methylation (H3K4me1, H3K4me3, and H3K36me3) at the IFN-γ promoter among the four groups at D180 (Figure 9), although a decreased trend was observed for a few histone marks. In order to determine whether the decrease in Ac-H3 and H3K4me1/3 may partially be due to an overall reduction of histone H3, we measured the total histone H3 and H4 levels in all four groups by western blot. As showed in Figure S3, there were no significant differences in total histone H3 and H4 levels in all four groups at D120 or D180. Spleen tissues from seven-day-old rats were then subjected to ChIP assays. Since these rats were still breast-feeding, only the VEH and DEX groups were studied. As shown in Figure S4, the VEH group showed large individual differences at D7. However, there were no differences in histone H3 acetylation (total H3, H3K9, and H3K14) and histone H3 lysine methylation (H3K4me1, H3K4me3, and H3K36me3) at the IFN-γ promoter between the VEH and DEX groups at D7. These results are compatible with the equal production of IFN-γ observed in the VEH and DEX groups at D7 [7].

Further evidences suggest that modifications of histones that lead to nucleosomal structural changes occur more often than in promoters regions [21]. For IFN-γ, modifications were detected at conserved noncoding sequences 1 (CNS1), CNS2, and Intron 3 [21,22,23]. Thus, in addition to the promoter site, we also determined the H3K9, H3K14 acetylation, and H3K36me3 levels at the CNS1, CNS2, and Intron 3 sites within the *ifng* locus of rat spleen with prenatal dexamethasone or postnatal HF diet exposure. Our data showed that there were no differences in H3K9 and H3K14 acetylation, and histone H3K36me3 status on the IFN-γ CNS1, CNS2, and Intron 3 sites among the four groups at D120 (Figure S5). However, there was a trend showing that the DHF group had lower enrichment for H3K14 acetylation at CNS1, CNS2, and Intron 3 sites at D120 than other groups (Figure S5B).

## 3. Discussion

In the present study, we used an animal model to study the impact of prenatal GC treatment plus postnatal HF diet on adaptive immunity. Our results suggest that a HF dietary environment from the postnatal stage exacerbates the potentially altered IFN-γ production triggered by prenatal GC treatment through site-specific histone modification at young adult stage. However, this impaired IFN-γ production and aberrant site-specific histone modification of the IFN-γ promoter had decreased by the middle-age adult stage. Although IL-10 was reported to inhibit the production of IFN-γ from lymphocytes [24], it cannot result from the decrease of IFN-γ by prenatal GC treatment plus a postnatal HF diet; this is because in our study, we found that IL-10 production increased in both the DEX and DHF groups at D120. In contrast to Th1 cytokines, Th2-related cytokines seem less susceptible to prenatal GC or postnatal HF exposure in adult rats.

Prenatal extreme stress has been correlated with high maternal corticosterone levels and lower birth weight [25]. In addition, high levels of endogenous or exogenous GC have been associated with an adverse metabolic profile, increased cardiovascular disease and altered cognitive function [25]. We previously found that prenatal dexamethasone exposure caused a reduction in the production of TNF-α rather than IFN-γ in adolescent male rat splenocytes [7]. O’Connor et al. reported that prenatal maternal anxiety could reduce IFN-γ production in infants at six months of age [26]. However, another study reported that prenatal dexamethasone exposure did not influence the production of IFN-γ in adolescent female rats [27]. Results from a small series of studies on infants whose mothers received immunosuppressants during pregnancy for the treatment of autoimmune disorders have also shown limited effects of prenatal corticosterone treatment on leukocyte sub-populations and IFN-γ production [5,28]. There are also conflicting results regarding the association between IFN-γ production and obesity. It is now generally accepted that chronic low-grade inflammation and activation of the immune system are involved in the pathogenesis of obesity-related insulin resistance [29]. A shift to a Th1 cytokine pattern of secreted cytokines has also been reported to contribute to inflammation in obesity [30]. Pacifico et al. reported that CD4-positive T cells secreted more IFN-γ in obese children than in controls [30]. Increased IFN-γ production has also been observed in obese mouse splenocytes upon stimulation with mitogen [12]. However, decreased IFN-γ production by mononuclear cells stimulated with PMA in obese subjects has also been reported [15]. In another report, mice fed a HF diet also showed lower IFN-γ production after NKT cell stimulation [31]. These inconsistent findings may be due to interactions between gender, age, developmental mode, methods of treatment, and even variations in the postnatal environment. Further studies are warranted to elucidate this issue.

Recent studies have suggested that many adult diseases begin in early life, both through in utero exposure and the postnatal environment, and relationships between prenatal stress, infant birth weight, and long-term adult consequences such as hypertension, metabolic disorders, and ischemic heart disease have been demonstrated [25,32,33]. Disease occurs due to genetic variations combined with external non-genetic factors. Epigenetics refers to functionally corresponding modifications of the genome that do not involve a change in nucleotide sequence. Epigenetic modifications include DNA methylation, histone modification (methylation, phosphorylation, and acetylation), chromatin remodeling, and microRNA regulation. Many environmental events can directly modify the epigenetic state of the genome during sensitive developmental periods, and one of the most important environmental factors is diet [34]. Prenatal programming most commonly occurs through epigenetic mechanisms, and DNA methylation is the most studied epigenetic marker. The relationship between prenatal GC treatment and DNA methylation has been well documented, and prenatal GC exposure has been shown to change organ-specific developmental trajectories of methylation in several organs [35,36]. These prenatal GC-induced changes in DNA methylation can persist into adulthood and even into the next generation [35]. This persistent DNA methylation provides a link between early life events and the subsequent risk of disease. The interaction between obesity and DNA methylation has also been well established [37,38]. In contrast, due to a greater complexity in study methods, histone modification has not been studied extensively in association with obesity or prenatal GC exposure, especially with regards to the immune system [7]. Evidence has suggested that GC treatment can exert a profound influence on the histone modifications and functioning of immune cells. Inhibition of HAT and recruitment of HDAC2 to inflammatory gene transcription sites were suggested as reasons behind the anti-inflammatory activity of steroids [39,40]. GC treatment was also shown to enhance activity of histone deacetylases (HDACs) 7, 9, 10, and 11 of mice splenic memory T cells [41]. However, a recent study showed that chronic GC treatment of natural killer cells (NK) can reduce NK cell granzyme B productions by reduction of histone promoter H4K8Ac as well as H3K9Ac while increasing IFN-γ and IL-6 production by increasing H4K8Ac/H3K27Ac as well as H3K4me3 in regulatory regions at the same time [42]. This dichotomous effect of GC on immune function seems to be dose- and time-dependent [42]. Thus deacetylation cannot totally explain the effects of GC treatment on histone modification. A study of site-specific histone modifications can provide us with a more precise mechanism for GC exposure. A HF diet could also lead to persistent change in chromatin accessibility with enriched H3K9me2 in mouse liver [43]. A HF diet has also been reported to cause histones H3 and H4 hypoacetylation, H3K4 hypomethylation, and increased HDACs 1, 2, 6 binding at the leptin promoter in adipose tissue [44]. However, the epigenetic effects of perinatal GC exposure and HF diet on the immune system are far from being fully understood. In a previous study, our group found that overexposure to prenatal DEX could decrease HDACs in the rat immune system [7]. Moreover, we found that prenatal dexamethasone exposure results in active chromatin signs (acetylation of histone H3 lysines, H3K4me1/3, and H3K36me3) and decreases in TNF-α promoter, leading to decreased TNF-α production [7]. Since the type and timing of exposure, sex of the child, and postnatal environment are all critical factors for prenatal programming [45], greater attention should be paid to evaluating prenatal programming. Although a previous study found that prenatal GC exposure may have a profound effect on the innate immune system rather than the adaptive immune system [7], our findings suggest a potentially altered adaptive immune function triggered by prenatal GC exposure. Our results demonstrated that prenatal dexamethasone and postnatal HF diet decrease IFN-γ production through site-specific histone modification.

Most of the reported histone modification profiles provide only a static picture linking certain modifications with active or inactive states. Our results also suggested that the immune impact of prenatal GC exposure and a postnatal HF diet was age-dependent. Similar age-dependent changes in IFN-γ production have been reported recently in prenatal immune-activated mice; however, the mechanism remains unknown [46]. Our results suggest that modification of chromatin structures with age is likely to be the major underling mechanism for the change in immune function. Age-associated changes in DNA methylation have been well documented; however, information regarding site-specific histone modification with age is still limited [47,48]. Kawakami et al. reported that H3 Lys9 acetylation decreased and H3 Ser10 phosphorylation increased with age in rat livers [42]; however, their study lacked functional validation. In the current study, we explored the IFN-γ production ability of rat splenocytes at newborn, young adult, and middle-age stages. Impaired IFN-γ production at the young adult stage was associated with decreases in H3K9 acetylation and H3K36me3 levels at the IFN-γ promoter with prenatal exposure to GC plus postnatal HF environment. Our findings suggest that kinetic changes in IFN-γ production ability with age are due to such epigenetic alternations. Clinically, obesity is an important risk factor for adolescent predisposition to immunosenescence and infection [49,50]. Thus, we speculate that prenatal stress plus postnatal HF diet may predispose young adults to IFN-γ-related health problems. One limitation of our study is that we do not analyze the cell populations in the spleen. Thus we did not know what kind of lymphocyte population is responsible for the age-dependent cytokine and epigenetic signature changes, although there were significant differences in blood leukocyte populations upon prenatal GC or postnatal HF diet exposure at D120. Further study with sorted splenocytes may provide us with better tools for interpretation.

It is worth noting that the effects of prenatal GC and/or postnatal HF diet exposure on blood leukocyte populations are also age-dependent. The T-cells’ development in the thymus is initialized by colonization of progenitor cells from the bone marrow into the thymus, where they undergo differentiation from double-negative to double-positive then to single-positive CD4 or CD8 T-cells [51]. Most mature T lymphocytes can be divided into CD4+CD8− helper cells or CD4−CD8+ cytotoxic cells. Only about 1% to 5% of the peripheral immature T-cells showed a CD4−CD8− double-negative (DN) or double-positive phenotype [52,53]. It has been reported that a decrease in thymic cellularity and mature T-cell output are observed on mice fed a HF diet [54]. Mice on a HF diet also showed disrupted T-cell maturation with increased double-negative and decreased single-positive thymocytes [54]. Our results agreed with this observation. In our study, both VHF and DHF showed higher CD4−CD8a− double-negative cells at D120. The impacts of prenatal GC and/or postnatal HF diet exposure on blood leukocyte populations disappeared at D180.

To address the complex interactions of genes with the environment, a three-hit concept of vulnerability and resilience has been developed [55]. This concept includes the interaction of genetic factors (hit 1) with early-life environmental factors (hit 2), altering gene expression and leading to phenotypes with susceptibility to later-life exposure (hit 3) (i.e., vulnerability); however, when exposed to another type of environment the same individual is expected to be resistant to the phenotypes (i.e., resilience). In our study, a HF dietary environment (hit 3) from the postnatal stage exacerbated the altered IFN-γ production triggered by prenatal GC exposure (hit 2) through site-specific modification at the adolescent stage. At the adult stage, the impaired IFN-γ production and aberrant histone modification on the IFN-γ promoter had decreased. Thus, “age” seems to protect rats from impaired IFN-γ production. However, aging is a process of genetic and epigenetic interactions at all biological levels, and epigenetic modification occurs during the aging process [56]. The elderly population has been reported to have impaired Th1 cell responses and IFN-γ production [57]. Thus, further research into immune programming at an advanced age is also needed to understand the long-term immune consequences of prenatal GC plus postnatal HF diet. The findings of this study may help to clarify the mechanism by which prenatal exposure to GC and the postnatal environment exert effects on fetal immunity programming.

## 4. Material and Methods

### 4.1. Animals

This study was performed according to the Guide for the Care and Use of Laboratory Animals of the National Institutes of Health and approved by the Institutional Animal Care and Use Committee of Chang Gung Memorial Hospital Kaohsiung Medical Center (IACUC No 2014062304 on 1 September 2014). Virgin Sprague-Dawley (SD) rats (about 12–16 weeks old) were obtained (BioLASCO Taiwan Co., Ltd., Taipei, Taiwan), and housed and maintained in a facility certified by the Association for the Assessment and Accreditation of Laboratory Animal Care International. The virgin SD female rats were allowed to mate with male rats for 24 h, and were then separated from the male rats and housed individually in a standard plastic home cage. Mating was confirmed by observation of a vaginal plug. After confirmation of pregnancy, the pregnant female rats were randomly assigned to two groups: the vehicle group and the dexamethasone exposure group. In the dexamethasone exposure group, daily intraperitoneal injections of dexamethasone (0.1 mg/kg/day) were given from a gestational age of 14–21 days. The vehicle group received daily intraperitoneal injections of normal saline during the same period. In both groups, 24 female rats were mated to deliver 10 litters. The subject offspring from litters of eight pups were standardized in terms of the received quantity of milk and maternal pup care after birth. In order to prevent maternal rejection, we did not weigh the pups at birth. Each offspring was left with the mother until weaning. Pups were weaned at postnatal day 21 and grouped three male animals per cage until testing in adulthood. The sex ratio of male to female offspring was approximately 1:2. The male offspring were then divided into four groups: the vehicle (VEH) group, prenatal dexamethasone exposure group (DEX), postnatal high-fat diet group (VHF), and prenatal dexamethasone exposure with postnatal high-fat diet group (DHF) (*n =* 6 for each group). Male offspring rats in the VEH and DEX groups received a control diet (protein 23.5%, fat 4.5%, crude fiber 5.0%, crude ash 7.0%, and water 13%; Fwusow Taiwan Co., Ltd., Taichung, Taiwan). Male offspring in the VHF and DHF groups received a HF diet (58% fat, Research Diet, D12331) from weaning to four or six months of age as indicated.

### 4.2. Experimental Procedures and Specimen Collection

The rats in all four groups were killed at postnatal day 120 (D120) or postnatal day 180 (D180) to assess the immunotoxicity of exposure to prenatal dexamethasone and a postnatal HF diet. One pup from each litter was sampled and assigned to an experimental group randomly to reduce the litter effects. Rats were euthanized using xylazine (50 mg/kg; Bayer, Taipei, Taiwan) and ketamine (50 mg/kg; United Biomedical, Hsinchu, Taiwan) at a 1:1 mixture by intramuscular injection. The body weight, thymus weight, and spleen weight were then recorded, and the spleens were used for further studies. Blood specimens were also collected for analysis.

### 4.3. Peripheral Blood Analysis and Plasma Immunoglobulin Detection

The specimens of blood were collected in heparin-rinsed tubes. Total blood cell count and white blood cell differential count were measured using a Sysmex XT-1800i system (Sysmex, Hyogo, Japan). For lymphocyte subset analysis, leukocytes were stained with the following antibodies: FITC-conjugated anti-rat CD3, PE-conjugated anti-rat CD45RA, APC-conjugated anti-rat CD4, and PerCP-conjugated anti-rat CD8a BD. All antibodies were purchased from BD Pharmingen (Sparks, NV, USA). Data were acquired using a FACS Aria I cytometer (Becton Dickinson, Franklin, NJ, USA) and analyzed using FlowJo software (Version 8.8, Tree Star, San Carlos, CA, USA).

### 4.4. Splenocyte Cultures and Drug Treatment

Splenocytes were separated from whole spleens as previously described [7]. In brief, the spleens were washed and pressed through a 30-µm nylon mesh. After lysis of contaminating red blood cells, the splenocytes (purity ≥ 90%; 2 × 10^6^ cells/mL) were cultured in 24-well plates in enriched RPMI 1640 medium. Cultured splenocytes were then stimulated with or without 100 ng/mL of lipopolysaccharides (LPS) or 5 μg/mL of concanavalin A (ConA) (Sigma, St. Louis, MO, USA). The culture supernatants and cell pellets were collected at indicated time points for further experiments. Viability of the cultured splenocytes was determined by Trypan blue staining before collection to ensure that there were no differences between the four groups.

### 4.5. 5-Bromo-2′-Deoxyuridine (BrdU) Cell Proliferation Assay

Proliferation of splenocytes was assessed using a BrdU assay as previously described [58]. In brief, splenocytes were suspended to 5 × 10^5^ cells/mL and stimulated with or without 5 μg/mL of ConA. After 48 h of incubation, BrdU reagent was added to the proliferating splenocytes allowing for labeling over the following 24 h. Proliferation was then measured using a BrdU assay (Millipore, Billerica, MA, USA) according to the manufacturer’s instructions. The results were presented as the ratio of optical density of ConA stimulation/optical density of phosphate-buffered saline (PBS) stimulation.

### 4.6. Cytokine Analysis

The cell culture supernatants were collected, and the innate and adaptive immunity cytokines were then detected using a Luminex 200 system (Luminex, Austin, TX, USA). Supernatant concentrations of IP-10, MCP-1, IL-1β, IL-2, IL-4, IL-5, IL-10, IL-13, TGF-β1, TGF-β2, and TGF-β3 were assessed using a Multiplex Assay (Millipore) system according to the manufacturer’s instructions. Cytokine levels were measured using xPONENT version 4.2 software (Luminex). TNF-α and IFN-γ production was detected using an ELISA assay (R & D Systems, Minneapolis, MN, USA).

### 4.7. Reverse Transcription (RT)-Polymerase Chain Reaction (PCR)

RT-PCR was performed as previously described [7]. In brief, total RNA was extracted from spleen tissues with Trizol reagent (Invitrogen, Carlsbad, CA, USA). A 5-µg sample of total RNA was reversed transcribed with 200 U of Moloney murine leukemia virus reverse transcriptase (Invitrogen). PCR was performed in 20 µL reaction volumes containing 2 µL of 1:10 diluted cDNA obtained from reverse transcribed RNA, specific primers, 2.5 mM MgCl, and Maxima SYBR Green/Fluorescein qPCR Master Mix (Thermo Scientific, Waltham, MA, USA). The cycling protocol composed of one cycle of 10 min at 95 °C followed by 45 cycles of denaturation for 10 s at 95 °C, annealing for 20 s at 55 °C, and then extension for 20 s at 72 °C. Primers were described in Table S1. Threshold cycles (*C*_t_) were detected with Light Cycler software (ver. 1.5.0, Roche, Mannheim, Germany). Standard curves were then plotted with *C*_t_ versus log cDNA quantity, and the quantities of the samples were obtained from the standard curves. The comparative *C*_t_ method was employed for relative quantification of gene expression. The averaged *C*_t_ was subtracted from the corresponding averaged peptidylprolyl isomerase B (PPIB) value for each sample, resulting in Δ*C*_t_. ΔΔ*C*_t_ was calculated by subtracting the average control Δ*C*_t_ value from the average experimental Δ*C*_t_ value. The fold increase was calculating as 2^−^^ΔΔ*C*t^ for the experimental vs. control samples.

### 4.8. Chromatin Immunoprecipitation (ChIP) Assay

ChIP assays were performed as previously described [7]. In brief, 50 mg of spleen tissue was cut into small pieces and fixed with warmed 1% formaldehyde (37 °C). The samples were then centrifuged and washed with ice-cold PBS. The ChIP assays were performed by using an EZ-Magna ChIP™ A kit (Millipore) according to the manufacturer’s instructions. DNA was sheared to an average length of 200–1000 bp by sonication, and then 5 μL of the supernatant was removed as the “Input” and saved at 4 °C. The supernatant was then incubated overnight at 4 °C with 5 μg of the indicated antibodies for immunoprecipitation. The antibodies used in the immunoprecipitation were as follows: acetyl histone H3 (Millipore), acetyl histone H3 lysine 4 (Cell Signaling, Danvers, MA, USA), monomethyl histone H3 lysine 4 (Abcam, Cambridge, UK), acetyl histone H3 lysine 9 (Cell Signaling), trimethylhistone H3 lysine 36 (Abcam), and trimethyl histone H3 lysine 4 (Abcam). The immunoprecipitated DNA was then eluted and quantitated by real-time PCR performed at an annealing temperature of 57 °C for 45 cycles. The primers used for the rat IFN-γ promoter and body are described in Table S1. *C*_t_ values of diluted input were adjusted to 100% of the input by subtracting 3.322 cycles (i.e., log2 of 10) from the *C*_t_ value of the diluted input. The amount of DNA precipitated by a specific antibody was calculated as the percentage of the input using the following formula: % of input = 2^Δ*C*t^ × 100, where Δ*C*_t_ = *C*_t input_ − *C*_t IP_ [59]. The results were expressed as fold over the control samples.

### 4.9. Western Blotting

Western blot was performed as previously described [60]. Each 50 mg of spleen tissue was homogenized with protein extraction solution (iNtRon biotechnology, Sungnam, Korea) according to the manufacturer’s instructions. After protein concentrations were determined, 50-µg samples were boiled and subjected to 12% SDS-PAGE for each lane. After transferring and blocking to a polyvinylidene difluoride (PVDF) membrane, the membrane was incubated for 2 h with anti-histone H3 (Santa Cruz Biotechnology, Santa Cruz, CA, USA) or H4 antibody (Santa Cruz) at 1:500. After washing and incubation for 2 h with peroxidase-labeled secondary antibody diluted 1:1000 in T-BST, The signal was obtained using a Bio-Rad Molecular Imager ChemiDocMP and quantified by Image Lab version 5.0 software (Bio-Rad, Richmond, CA, USA).

### 4.10. Statistics

For most parameters, statistical analysis was performed using a Mann–Whitney U test; otherwise, ANOVA with the Bonferroni post hoc test for multiple comparisons of two or four groups was used as indicated. Data are expressed as the mean ± standard error of the mean. A *p*-value of less than 0.05 was defined to be statistically significant for all tests. Statistical tests for all analyses were performed using with SPSS 15.0 for Windows XP (SPSS, Inc., Chicago, IL, USA).

## 5. Conclusions

Prenatal dexamethasone and a postnatal HF diet decreased IFN-γ production through a site-specific and an age-dependent histone modification. These findings suggest a mechanism by which prenatal exposure to GC and a postnatal environment exert effects on fetal immunity programming.

## Figures and Tables

**Table 1:** Body weight, immune organs weight, and ratio of immune organs weight to body weight in male offspring received prenatal dexamethasone or/with a postnatal high-fat diet at postnatal day 120 and 180. The data are presented as mean ± S.E.M. (*n =* 6 for each group). Only significant *p*-values as compared with the VEH group are listed here.

BW/Immune Organ Weight at D120				
Group	VEH	DEX	VHF	DHF
BW (g)	497.60 ± 40.40	500.17 ± 19.93	646.00 ± 19.93 (*p =* 0.006)	568.50 ± 34.45
Thymus (g)	0.34 ± 0.03	0.40 ± 0.04 (*p =* 0.046)	0.39 ± 0.04	0.36 ± 0.04
Spleen (g)	0.79 ± 0.10	0.91 ± 0.03 (*p =* 0.002)	0.92 ± 0.03 (*p =* 0.016)	0.88 ± 0.05
Thymus/BW	(4.99 ± 0.07)× 10^−4^	(8.98 ± 0.60) × 10^−4^ (*p =* 0.033)	(6.14 ± 0.81) × 10^−4^	(6.39 ± 0.08) × 10^−4^
Spleen/BW	(1.52 ± 0.08) × 10^−3^	(1.74 ± 0.07) × 10^−3^ (*p =* 0.045)	(1.43 ± 0.05) × 10^−3^ (*p =* 0.039)	(1.55 ± 0.04) × 10^−3^ (*p =* 0.049)
**BW/Immune Organ Weight at D180**				
**Group**	**VEH**	**DEX**	**VHF**	**DHF**
BW (g)	649.71 ± 22.93	691.11 ± 26.03	751.67 ± 29.90 (*p =* 0.046)	943.38 ± 49.43 (*p* < 0.001)
Thymus (g)	0.27 ± 0.02	0.20 ± 0.02	0.34 ± 0.04 (*p =* 0.044)	0.31 ± 0.04
Spleen (g)	1.01 ± 0.05	1.06 ± 0.07	1.01 ± 0.05	1.24 ± 0.06 (*p =* 0.014)
Thymus/BW	(4.11 ± 0.31) × 10^−4^	(2.91 ± 0.17) × 10^−4^ (*p =* 0.049)	(4.56 ± 0.56) × 10^−4^	(3.34 ± 0.38) × 10^−4^
Spleen/BW	(1.55 ± 0.06) × 10^−3^	(1.56 ± 0.09) × 10^−3^	(1.35 ± 0.07) × 10^−3^	(1.33 ± 0.08) × 10^−3^

**Table 2:** Blood cell profile and white blood cell classification in male offspring received prenatal dexamethasone or/with a postnatal high-fat diet at postnatal day 120 and D 180. The result is presented as mean ± S.E.M. (*n =* 6 for each group). Only significant *p*-values as compared with the VEH group are listed here.

D120				
Group	VEH	DEX	VHF	DHF
WBC (10^3^/μL)	12.16 ± 2.49	10.57 ± 1.56	10.57 ± 0.94	8.58 ± 0.89
RBC (10^6^/μL)	9.26 ± 0.24	9.45 ± 0.26	9.14 ± 0.21	9.07 ± 0.17
HGB (g/dL)	16.38 ± 0.44	15.44 ± 0.45	15.98 ± 0.32	15.33 ± 0.23
PLT (10^3^/μL)	1036.50 ± 51.86	1075.60 ± 191.95	1100.67 ± 70.79	935.00 ± 51.41
Neutrophil (%)	12.00 ± 0.24	17.34 ± 1.67 (*p =* 0.014)	22.33 ± 3.45 (*p =* 0.018)	19.08 ± 2.74 (*p =* 0.033)
Lymphocyte (%)	84.05 ± 0.35	78.66 ± 1.82 (*p =* 0.014)	73.78 ± 3.57 (*p =* 0.028)	76.32 ± 3.14
Monocyte (%)	2.33 ± 0.30	2.68 ± 0.47	2.85 ± 0.37	3.65 ± 0.47 (*p =* 0.049)
Eosinophil (%)	1.58 ± 0.41	1.28 ± 0.45	1.00 ± 0.18	0.92 ± 0.18
Basophil (%)	0.05 ± 0.03	0.04 ± 0.02	0.03 ± 0.02	0.03 ± 0.03
CD4+ (%)	62.08 ± 3.77	60.02 ± 1.99	58.97 ± 2.54	55.72 ± 1.71
CD8a+ (%)	36.06 ± 3.72	37.37 ± 2.53	36.68 ± 2.54	40.93 ± 1.74
CD4+CD8a+ (%)	1.50 ± 0.21	2.20 ± 0.67	3.65 ± 0.85 (*p =* 0.042)	2.72 ± 0.69
CD4−CD8a− (%)	0.32 ± 0.04	0.40 ± 0.08	0.67 ± 0.13 (*p =* 0.014)	0.62 ± 0.06 (*p =* 0.032)
CD45RA (%)	2.40 ± 0.65	1.15 ± 0.40	1.28 ± 0.39	1.68 ± 0.70
**D180**				
**Group**	**VEH**	**DEX**	**VHF**	**DHF**
WBC (10^3^/μL)	11.55 ± 1.98	11.79 ± 1.33	7.82 ± 0.86	7.06 ± 0.76 (*p =* 0.030)
RBC (10^6^/μL)	9.16 ± 0.31	9.52 ± 0.33	9.04 ± 0.11	8.92 ± 0.17
HGB (g/dL)	15.35 ± 0.51	16.68 ± 0.63	15.07 ± 0.18	16.30 ± 0.30
PLT (10^3^/μL)	1070.75 ± 158.53	1215.40 ± 112.81	1137.33 ± 51.84	1217.50 ± 20.33
Neutrophil (%)	19.35 ± 3.44	18.43 ± 0.77	20.75 ± 2.92	19.27 ± 2.22
Lymphocyte (%)	75.88 ± 3.54	77.83 ± 1.31	61.00 ± 10.85	76.70 ± 2.33
Monocyte (%)	3.30 ± 0.46	2.83 ± 0.34	4.67 ± 0.54	3.25 ± 0.38
Eosinophil (%)	1.45 ± 0.44	0.90 ± 0.23	1.12 ± 0.12	0.78 ± 0.23
Basophil (%)	0.03 ± 0.03	0.04 ± 0.02	0.02 ± 0.02	0.00 ± 0.00
CD4+ (%)	66.03 ± 2.25	66.06 ± 2.04	58.75 ± 2.71	62.83 ± 1.50
CD8a+ (%)	31.80 ± 1.98	32.60 ± 2.08	39.78 ± 2.58	35.60 ± 1.58
CD4+CD8a+ (%)	0.73 ± 0.07	0.64 ± 0.07	0.60 ± 0.09	0.57 ± 0.08
CD4−CD8a− (%)	1.43 ± 0.42	0.74 ± 0.05	0.88 ± 0.07	0.98 ± 0.19
CD45RA (%)	1.28 ± 0.23	2.22 ± 0.67	1.03 ± 0.18	1.22 ± 0.28

## Supplementary Materials

Supplementary materials can be found at www.mdpi.com/1422-0067/17/10/1610/s1.
