# Supplementary material for: Prenatal Dexamethasone and Postnatal High-Fat Diet Decrease Interferon Gamma Production through an Age-Dependent Histone Modification in Male Sprague-Dawley Rats
Source: Int J Mol Sci. 2016 Sep 22;17(10):1610. doi: 10.3390/ijms17101610 (PMC5085643; doi:10.3390/ijms17101610)
Supplement: Supplementary file 1 [file ijms-17-01610-s001.pdf]

Supplementary Materials: Prenatal Dexamethasone and Postnatal High-Fat Diet Decreases Interferon Gamma Production through an Age-Dependent Histone Modification in Male Sprague-Dawley Rats

Hong-Ren Yu, You-Lin Tain, Jiunn-Ming Sheen, Mao-Meng Tiao, Chih-Cheng Chen, Ho-Chang Kuo, Pi-Lien Hung, Kai-Sheng Hsieh and Li-Tung Huang

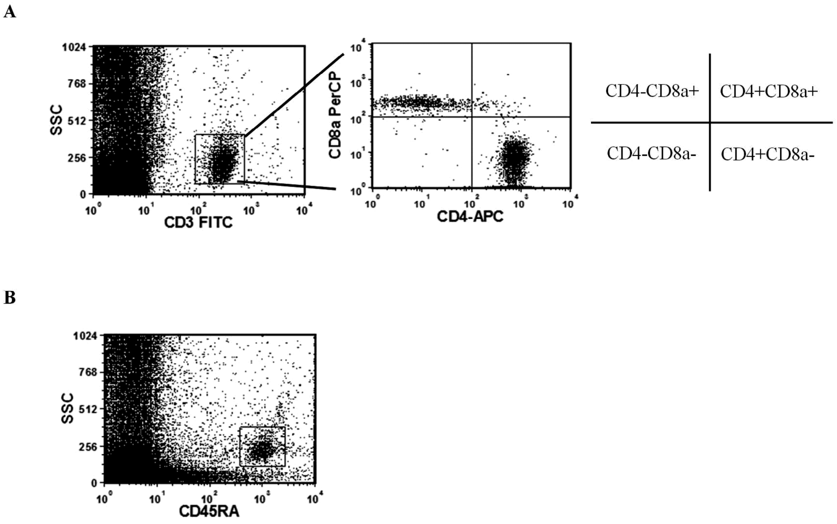

Figure S1. FACS dot plot represents the CD4/8a and CD45RA staining in the indicated gated cells. (**A**) For T lymphocyte subset analysis, CD3-positive cells were gated and further analyzed for CD4 and CD8a as indicated; and (**B**) For B lymphocyte analysis, CD45RA-positive cells were gated and analyzed.

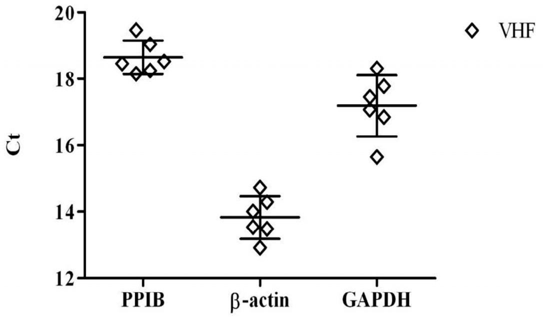

	Mean ± SD	%CV	N
**PPIB**	**18.650 ± 0.511**	**2.738**	**6**
**β-actin**	**13.830 ± 0.642**	**4.641**	**6**
**GAPDH**	**17.188 ± 0.916**	**5.332**	**6**

Figure S2. The expressions of indicated reference gene mRNA in rat spleen. Two micrograms of cDNA was used for each sample and indicated for PCR with PPIB, β-actin, or GAPDH primers. Among the three reference genes, PPIB had the smallest coefficient of variance (CV). N = case number.

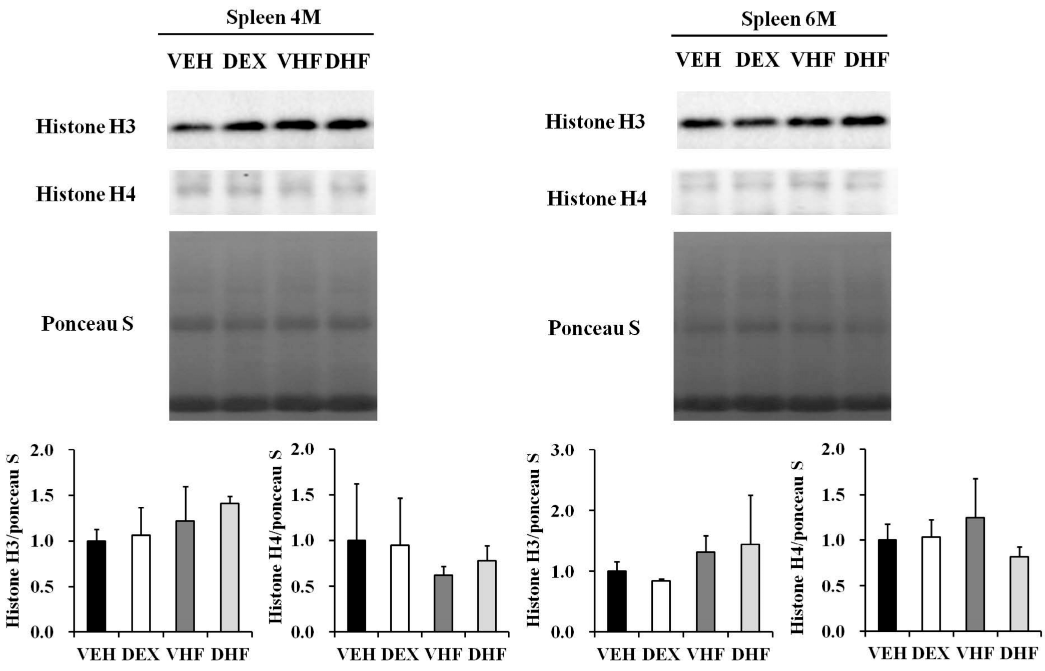

Figure S3. Western blot of total abundances of histone H3 and H4 in rat spleens at day 120 and day 180. Fifty mg of spleen tissue sample was homogenized and indicated for each lane. Both total histone H3 and total histone H4 were quantified with Ponceau staining. Data presented were obtained from three replicated experiments. Abbreviation: VEH; vehicle: DEX; prenatal dexamethasone exposure: VHF; postnatal high-fat diet exposure: DHF; prenatal dexamethasone plus postnatal high-fat diet exposure: 4M; 4 month-old; 6M; 6 month-old.

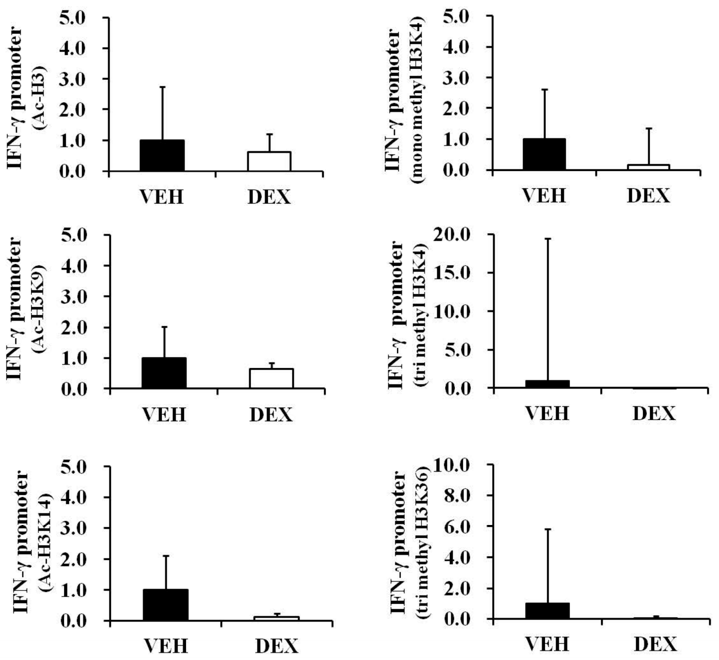

Figure S4. Histone H3 lysine acetylation and methylation levels at the IFN-γ promoter of spleens with/without the indicated treatment at D7. Bar graphs showing the total H3 lysine, H3K9 and H3K14 acetylation, and H3K4me1, H3K4me3, and H3K36me3 levels of the indicated treatment group relative to the vehicle at the IFN-γ promoter. The results were expressed as fold difference over the vehicle (mean ± SEM; n = 3).

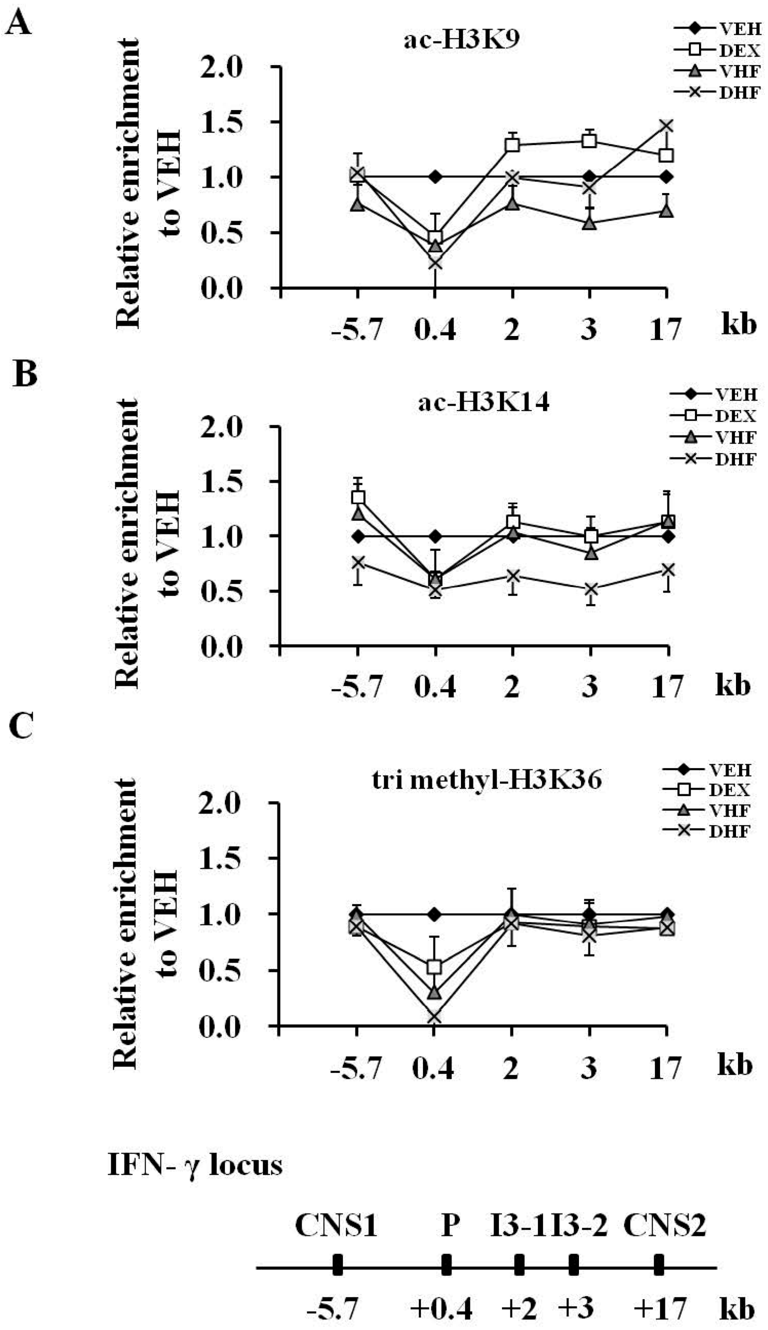

Figure S5. Histone modifications within the Ifng locus of rat spleen tissues. Chromatin of spleen tissues from D120 rats was immunoprecipitated by using (**A**) anti-acetyl-histone H3 lysine 9 (ac-H3K9); (**B**) anti-acetyl-histone H3 lysine 14 (ac-H3K14); or (**C**) anti-trimethyl-histone H3 lysine 36 (H3K36me3) antibodies. Intron 3 was analyzed with two different primer sets. The x-axis shows the Ifng locus with positions numbered relative to the transcription start site. The black boxes represent CNS1, promoter (P), third intron (I3), and CNS2. The results were expressed as fold difference over the vehicle (mean ± SEM; n = 3–5). The statistical differences at the promoter site are as shown in Figure 7.

Table S1. The primer sequences used for quantitative polymerase chain reaction (qPCR) and chromatin immunoprecipitation (ChIP) assay.

Target Gene	Primer	Primer Sequences for qPCR
IL-8	sense	5′-TGCACCCAAACCGAAGTCATAGCC-3′
	antisense	5′-GCGTTCACCAGACAGACGCCA-3′
IL-6	sense	5′-TGCCTTCTTGGGACTGATGTTG-3′
	antisense	5′-TGGTCTGTTGTGGGTGGTATCC-3′
TNF-α	sense	5′-GGCTGCCCCGACTACGT-3′
	antisense	5′-AGGGCAAGGGCTCTTGATG-3′
T-bet	sense	5′-TCCACCCAGACTCCCCAACA-3′
	antisense	5′-GGCTCACCGTCATTCACCTCCA-3′
PPIB	sense	5′-CTGTCGATTCCCTCACAGGT-3′
	antisense	5′-AAAATCAGGCCTGTGGAATG-3′
β-actin	sense	5′-TACTGCCCTGGCTCCTA-3′
	antisense	5′-GGGCCGGACTCATCGTA-3′
GAPDH	sense	5′-TCTTGTGCAGTGCCAGCCTC-3′
	antisense	5′-GTCACAAGAGAAGGCAGCCCTGG-3′
**Target Gene**	**Primer**	**Primer Sequences for ChIP assay**
IFN-γ promoter	sense	5′-TCGGTGAGGTGTTCGTTGAC-3′
	antisense	5′-AAGAATGAAAACCATGAAGG-3′
IFN-γ CNS1	sense	5′-TCAGGAGTTCCAGGAGTTGAACCA-3′
	antisense	5′-TCCTCCAGCCCTTCAGGCAGATTT-3′
IFN-γ CNS2	sense	5′-AGGATGCCCTGTGAGTCACTGA-3′
	antisense	5′-CACTGGTTGAAAGTCACAGGAACT-3′
IFN-γ intron 3-1	sense	5′-AATCGGGCTCTGAGGAGACT-3′
	antisense	5′-TGAGCTGCATAGCACGAGAG-3′
IFN-γ intron 3-2	sense	5′-CCCATCCCAGAAATGATGAC-3′
	antisense	5′-TCTACCAAGGAGCCCAGAGA-3′

Abbreviation: PPIB: vehicle peptidylprolyl isomerase B; CNS: conserved noncoding sequence.
